# Normotensive metabolic syndrome in Transient Receptor Potential Canonical Channel type 1 *Trpc1^−/−^* mice.

**DOI:** 10.1242/bio.060280

**Published:** 2024-07-31

**Authors:** Richard Matthew Atkins, Meghan Pantalia, Christopher Skaggs, Alexander Ku Lau, Muhammad Bilal Mahmood, Muhammad Mubeen Anwar, Lindsay Barron, Bonnie Eby, Usman Khan, Leo Tsiokas, Kai Lau

**Affiliations:** ^1^ Department of Medicine, University of Oklahoma Health Sciences Center, Oklahoma City, OK 73104, USA; ^2^ Department of Cell Biology, University of Oklahoma Health Sciences Center, Oklahoma City, OK 73104, USA; ^3^ Division of Nephrology, Department of Medicine, University of Oklahoma Health Sciences Center, Oklahoma City, OK 73104, USA

**Keywords:** TPRC1, Metabolic syndrome, Calcium channel, Diabetes, Ultrasound, Animal model

## Abstract

Metabolic syndrome has become a global epidemic, affecting all developed countries and communities with growing economies. Worldwide, increasing efforts have been directed at curbing this growing problem. Mice deleted of the gene encoding Type 1 Transient Receptor Potential Canonical Channel (*Trpc1*) were found to weigh heavier than controls. They had fasting hyperglycemia and impaired glucose tolerance compared with wild-type controls. Beyond 1 year of age, plasma triglyceride level in *Trpc1^−/−^* mice was elevated. Plasma cholesterol levels tended to be higher than in controls. The livers of *Trpc1^−/−^* mice were heavier, richer in triglyceride, and more echogenic than those of controls on ultrasound evaluation. Hematocrit was lower in *Trpc1^−/−^* mice of both genders beginning at the second to third months of age in the absence of bleeding or hemolysis. Measured by the indirect tail-cuff method or by the direct arterial cannulation, blood pressures in null mice were lower than controls. We conclude that TRPC1 gene regulates body metabolism and that except for hypertension, phenotypes of mice after deletion of the *Trpc1* gene resemble mice with metabolic syndrome, suggesting that this could be a good experimental model for future investigation of the pathogenesis and management of this disorder.

## Teaser

**Summary:** *Trpc1-*knockout mice are obese, diabetic, hypertriglyceridemic and anemic with liver steatosis on ultrasound, providing a model for studying the pathogenesis and management of metabolic syndrome, a growing global epidemic.

## INTRODUCTION

Metabolic syndrome has become increasingly recognized in clinical medicine, currently defined as displaying key components of systemic hypertension, glucose intolerance, dyslipidemia, obesity, and liver disease (Huang, 2009; Kennedy et al., 2010; Wong et al., 2016). It is associated with increased cardiovascular risks, prompting urgent therapeutic interventions, although largely empirical at present. Currently, neither the precise pathogenesis nor the rational management have been defined, despite active ongoing research. One obstacle relates to the paucity of established animal models. Another impediment is the absence of a testable hypothesis in either the pathogenesis or the treatment.

The superfamily of TRP channels consists of a large number of cation channels that are generally permeable to monovalent and divalent cations. The 28 mammalian TRP channels are currently grouped into six main subfamilies: the TRPC (canonical), TRPV (vanilloid), TRPM (melastatin), TRPP (polycystin), TRPM (mucolipin), and the TRPA (ankyrin). Expressed in virtually every tissue and every cell type, TRP channels have been postulated to play a key role in the regulation of many cell functions (Nilius et al., 2007; Abramowitz and Birnbaumer, 2009; Onopiuk et al., 2020; Nesin and Tsiokas, 2014).

Each TRP channel subunit is made up of six putative transmembrane spanning segments (S1–6), a pore-forming loop between S5 and S6, and intracellularly located NH2 and COOH termini. Assemblage of channel subunits as homo- or hetero-tetramers generates various cation-selective channels. Indeed, several TRPCs, including TRPC-1, −4, and −5, can form heterotetramers, and the current properties are significantly different between TRPC5- and TRPC1/TRPC5-expressing cells. To date, major scientific advances have been generated, broadening our present understanding on the physiology and pathophysiology of TRP channels in human diseases (Beech et al., 2003; Beech, 2005; Ambudkar, 2007).

At present, only a few disorders of ion channels have been identified in which defects in *Trp* genes are the unequivocal cause of cellular dysfunction. TRPC1 was the first mammalian TRP channel to be recognized. Transient receptor potential *Trp* gene was first cloned in *Drosophila* (Montell and Rubin, 1989). TRPC1, a human homolog of the *Drosophila* store-operated channel was identified (Wes et al., 1995; Zhu et al., 1995) in the search for similar channel proteins in humans. TRPC1 and TRPC3 are the founding members of this ion channel family. The TRPC superfamily includes various channels involved in signal transduction. Dysfunctions in some members have been implicated in obesity, hypertriglyceridemia, diabetes and hypertension. For example, *TRPC1* expression was found to be reduced in diabetes (Zhang et al., 2009) but any causal relationship was obscure, although TRPC1 was the first TRP channel cloned and identified in pancreatic beta cells (Zhang et al., 2009).

Salivary gland fluid secretion has been reported to be impaired in mice deleted of the TRPC1 gene, presumably due to reduced store operated Ca (SOC) entry (Liu et al., 2007). SOC entry has been demonstrated to be a key component of the insulin secretion machinery (Sabourin et al., 2015). TRPC1 and the nuclear receptor, hepatic nuclear factor 4 alpha (HNF4 alpha), have been proposed as possibly involved in diabetic nephropathy (Niehof and Borlak, 2008), because *TRPC1* expression was found to be reduced in this condition, in 12-week-old, and in 26-week-old diabetic db/db mice. However, this had also been suggested as a late phenomenon (Niehof and Borlak, 2008).

In 20 individuals with diabetes and 26 people without diabetes undergoing coronary bypass surgery, saphenous veins were collected and vasomotor function was studied. RT-PCR and Western blotting analyses were also performed. Protein expression was found to be reduced for TRPC1 and TRPC6 in diabetic patients (Chung et al., 2009). It was reported that *TRPC1* genetic polymorphism was associated with type 2 diabetes in the Han Chinese population (Chen et al., 2013). One study suggested that TRPC1 is elevated in a porcine model of metabolic syndrome produced by feeding 6- to 9-month-old pigs a high-fat diet (Hu et al., 2009), but no causal relationship was suggested or defined. Recently it was shown that TRPC1 inhibited exercise-induced protection against obesity produced by a high-fat diet and in type 2 diabetes (Krout et al., 2017). However, it is unclear if the control animals were well matched to make such an interpretation unequivocal.

TRPC1/5 has been demonstrated to block adiponectin and create insulin resistance (Ahern, 2013; Sukumar et al., 2012). The ubiquitous expression of TRPC1 channels implies possible potential protection in many tissues or organs, even if the affected protein is mutated, absent or dysfunctional. The ability for TRPC channels to form potentially functional hetero-tetramers amid different members of a similar family suggests substantial system redundancy, conceivably to defend against serious organ dysfunctions despite minimal mutation or even complete loss of a given channel protein. Erasure of the TRPC1 gene is expected to cause multiple, though mild, organ dysfunctions. The *TRPC* superfamily comprises various cation channels shown to be involved in signal transduction, cell activation, and/or metabolism. Some members have been implicated in or linked to obesity, hypertriglyceridemia, diabetes and hypertension, four conditions currently being considered to be key features of the metabolic syndrome.

*TRPC1* was the first *TRPC* channel cloned and identified in the pancreatic beta-cells. Its expression has been shown to be reduced in diabetes, but a cause-and-effect relationship, if any, has not been shown or defined. To the best of our knowledge, the phenotypes, if any, of *Trpc1^−/−^* mice have neither been well described nor systematically evaluated. Recently, a role of TRP channels in metabolic syndrome has been proposed (Liu et al., 2008). Here, we tested the hypothesis that TRPC1 deficiency impairs glucose tolerance and causes abnormal lipid metabolism. If proven true, we would proceed to evaluate any possible organ dysfunctions.

## RESULTS

### Metabolic data

**Fig. 1.**
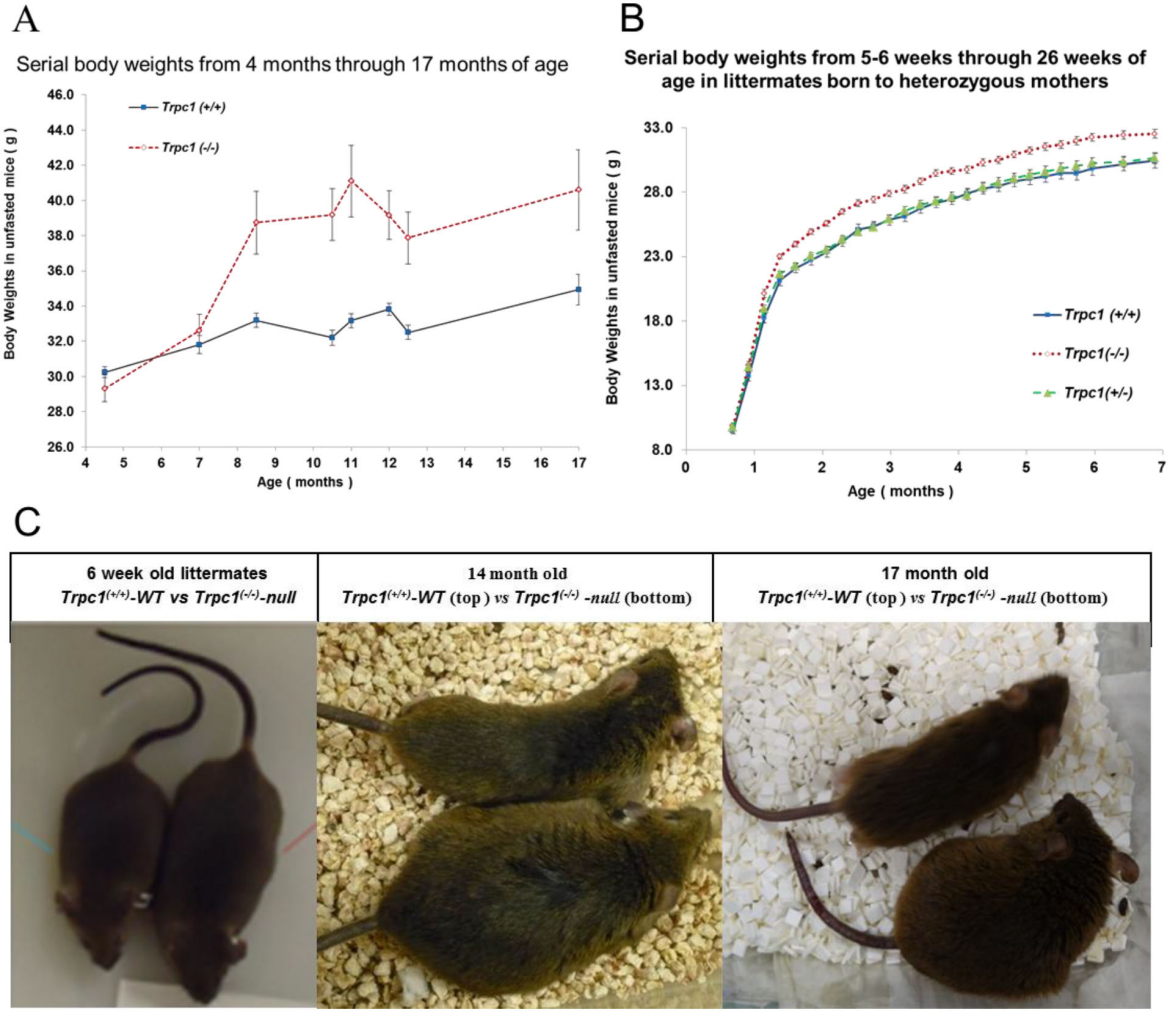
***Trpc1*^−/−^ mice are heavier.** (A) In one group displaying increased calorie intake, we measured serial body weights in wild-type (*n*=11) and *Trpc1*^−/−^ mice (*n*=12) from the 4th through the 17th months of age. We observed noticeable and significant (*P*<0.05 to <0.001) obesity beginning at the 8th month in *Trpc1*^−/−^ mice vs *Trpc1*^+/+^ wild type. (B) Obesity in male *Trpc1*^−/−^ mice (*n*=22–45) was documented from the 5–6th weeks of age through the 26th week compared to *Trpc1*^+/+^ wild type (*n*=20–40) and *Trpc1*^+/−^ heterozygote littermates (*n*=10–63). *P*<0.001, *Trpc1^−/−^* vs wild type, 4th through 30th week; *P*<0.03, *Trpc1^−/−^* vs heterozygotes, 5th through 30th week; not significantly different between the wild type and the heterozygotes. (C) At 6 weeks, there was a difference in body morphology between littermate *Trpc1*^+/+^ mice and *Trpc1^−/−^* mice that were born to a common mother excluding maternal influence. *Trpc1^−/−^* male mice also displayed morphological differences from age-matched *Trpc1*^+/+^ mice purchased from Taconic lab at 14 months of age or purchased from Jackson labs at 17 months of age.

At 9 weeks of age, male *Trpc1^−/−^* mice exhibited a 20% heavier body weight, 28.3±0.5 g, *n*=8 vs 23.5±0.8 g, *n*=7 compared to age-matched wild-type controls, *Trpc1^+/+^* (*P*<0.001). In a larger subset, weight was heavier in *Trpc1^−/−^* mice beginning at 8 months of age and remained so through the 17th month, the last point of observation (Fig. 1 A,C). In littermates born to common heterozygous mutant mothers, the heavier weight in homozygous mutant mice over the wild type was apparent from the first month (Fig. 1B,C). At 10 months of age, the average food intake [3.3±0.3 vs 1.4±0.2 g/day of Purina rodent chow 5001 (3.36 Kcal/g), *P*<0.001] in homozygous mutant mice was 130% greater than that consumed by the wild-type controls. Fluid intake of sugar-containing cocktail (0.3 Kcal/ml) was not different (27.2±2.9 vs 23.7±2.9 ml/day). Mean total caloric (chow plus sugar-containing cocktail) intake was 62% greater in *Trpc1^−/−^* mice than control (19.3 vs 11.9 kcal/day), *P*<0.001.

### Glucose intolerance in *Trpc1^−/−^* mice

At 9 weeks of age, non-fasting blood glucose was 171±14 mg/dl in *Trpc1^−/−^* mice (*n*=8) vs 98±6 mg/dl in wild type (*n*=7), displaying a 75% greater difference (*P*<0.001). *Trpc1^−/−^* mice had shown persistently impaired blood glucose since 8.5 months of age. Mean blood glucose in 7 *Trpc1^−/−^* mice was 135±5 vs 100±3 mg/dl in 12 wild-type controls at 8.5 months (*P*<0.001). Blood glucose in five *Trpc1^−/−^* mice was 136±5, vs 89±4 mg/dl in 11 wild-type control mice at 10.5 months (*P*<0.001). It was 153±9 mg/dl in 12 *Trpc1^−/−^* mice vs 121±6 mg/dl in 11 wild*-*type control mice at 17 months. Non-fasting blood glucose was elevated in both male and female *Trpc1^−/−^* mice (Tables 1,2).

**Table 1.**
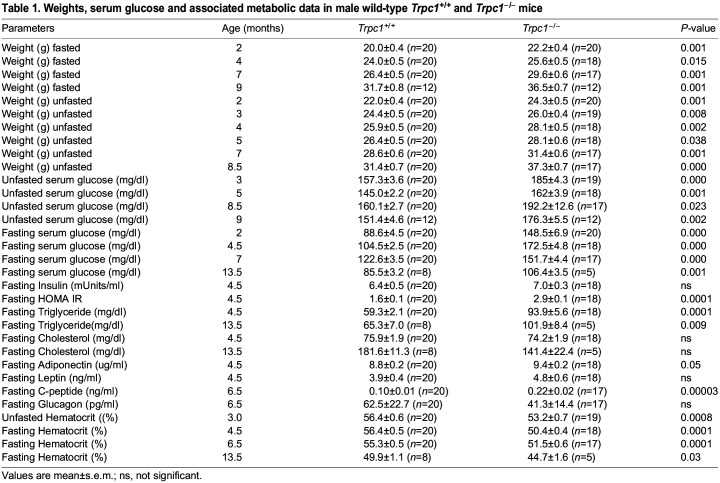
Weights, serum glucose and associated metabolic data in male wild-type *Trpc1^+/+^* and *Trpc1^−/−^* mice

**Table 2.**
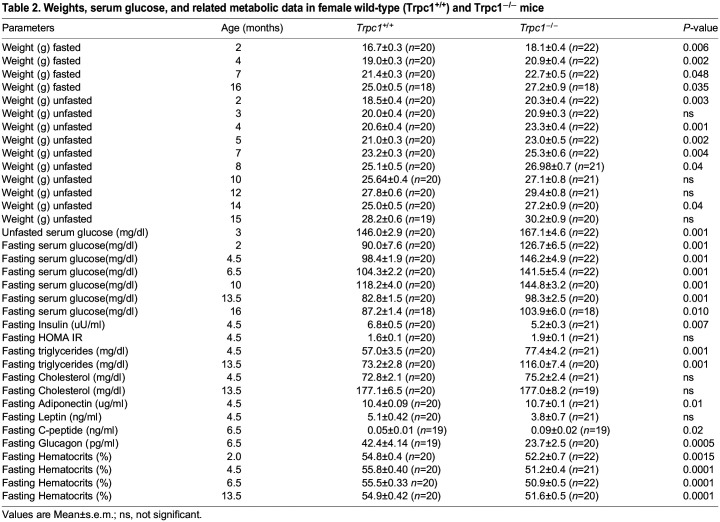
Weights, serum glucose, and related metabolic data in female wild-type (Trpc1^+/+^) and Trpc1^−/−^ mice

### Elevated fasting blood glucose levels in *Trpc1^−/−^* mice of both genders

As early as 1 month of age, blood glucose was elevated in *Trpc1^−/−^* mice (71.3±2.3 mg/dl, *n*=64) vs littermate wild-type control mice (63.5±3.1 mg/dl, *n*=40), *P*<0.05. Fasting blood glucose value in littermate *Trpc1^+/−^* mice (70.3±4.1 mg/dl, *n*=28) tended to be higher, but they were not significantly different from either the null mice or wild-type control mice. At 10.5 months of age, blood glucose was elevated in *Trpc1^−/−^* mice (109±8 mg/dl, *n*=5) vs wild-type control mice (72±5 mg/dl, *n*=11), *P*<0.001, and 12 months of age, blood glucose was elevated in *Trpc1^−/−^* mice (125±8 mg/dl, *n*=12) vs wild-type control mice (67±3 mg/dl, *n*=11), *P*<0.001. At 15.5 months of age, fasting blood glucose in null mice continued to be higher than littermate wild-type controls (65±2, *n*=28 vs 57±2 mg/dl *n*=15, *P*<0.02). Fasting blood glucose in littermate heterozygotes (61±3 mg/dl, *n*=15) was not statistically different from wild-type or *Trpc1^−/−^* mice (data not shown). Both homozygous mutant males and homozygous mutant females had significantly elevated fasting blood glucose levels from the first through the 16th month of age (Tables 1,2).

### Glucose tolerance test (GTT)

**Fig. 2.**
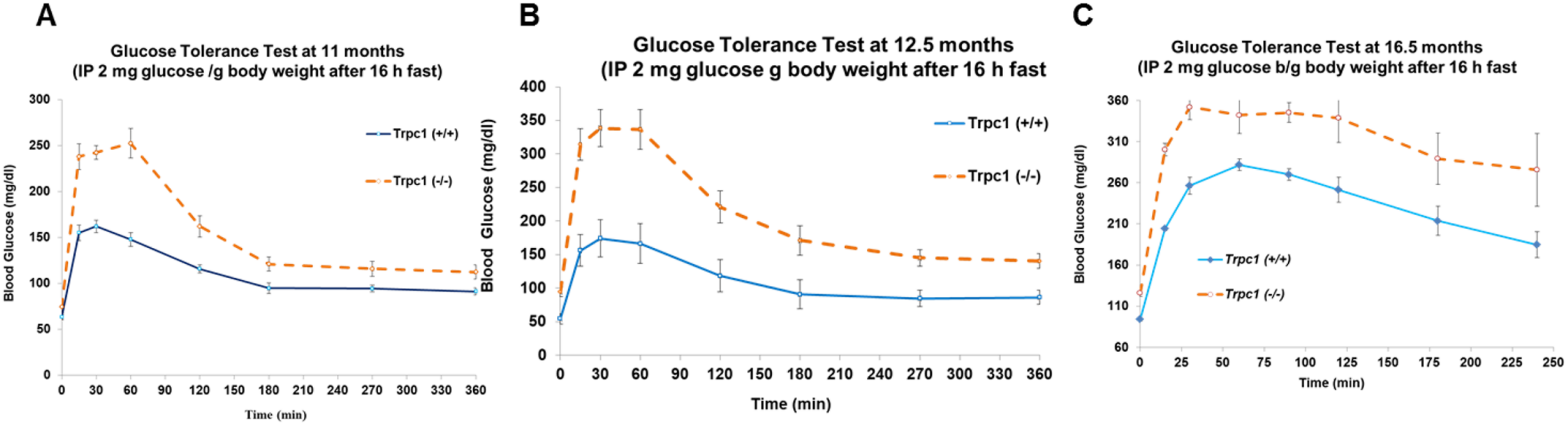
***Trpc1*^−/−^ mice have decreased glucose tolerance.** (A) Glucose tolerance tests in *Trpc1*^−/−^ mice (*n*=5) compared to *Trpc1*^(+/+)^ mice (*n*=12) at 11 months of age. (B) Glucose tolerance tests in *Trpc1*^(−/−)^ mice (*n*=11) compared to *Trpc1*^(+/+)^ mice (*n*=12) at 12.5 months of age. (C) Glucose tolerance tests in *Trpc1*^−/−^ mice (*n*=4) compared to *Trpc1*^(+/+)^ mice (*n*=8) at 16.5 months of age (please see Table 3 for numerical data).

At 11 months of age, GTT was performed by intraperitoneal injection of glucose (2 mg glucose/g body weight), given after a 16 h of fasting. Blood glucose was measured every 30–60 min. *Trpc1^−/−^* mice had abnormal glucose tolerance (*P*<0.001) compared with control. At 12.5 months of age, during the glucose tolerance test, the blood glucose values in *Trpc1^−/−^* mice were higher than in controls and were statistically significant at all time points. Thus at 12.5 months, *Trpc1^−/−^* mice had features of the metabolic syndrome, whereas the glucose tolerance test at 11 months showed elevated values in *Trpc1^−/−^* mice only during the first 2 h, with similar readings to wild-type controls thereafter (Fig. 2A,B). The minor difference in results between 11 and 12.5 months could reflect a milder defect in glucose intolerance at an earlier age. To confirm this interpretation, we tested glucose tolerance again at 16.5 months of age (Fig. 2C). At this time, the older *Trpc1^−/−^* mice again reproduced the glucose intolerance exhibited at 12.5 months (Table 3).

**Fig. 3.**
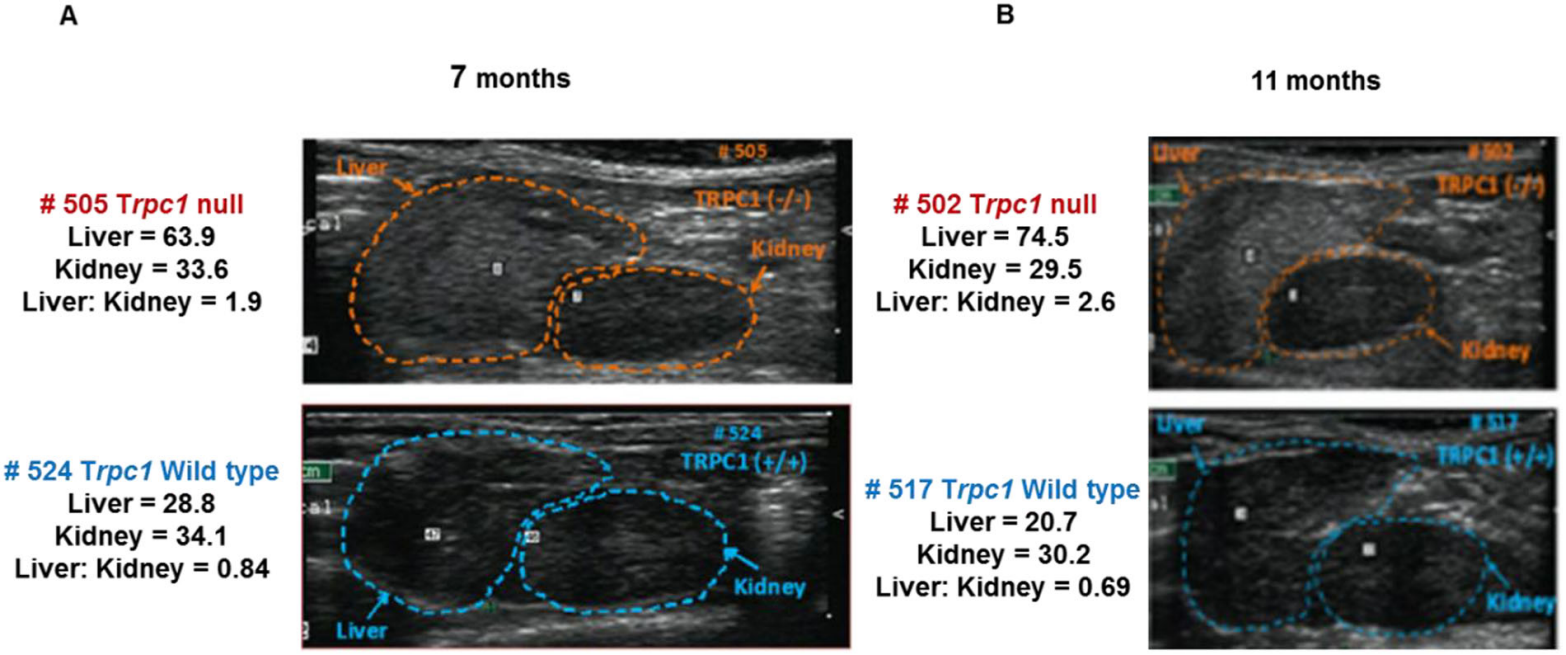
***Trpc1*^−/−^ mice show increased liver density.** Representative examples showing the difference in ultrasonic density between *Trpc1^−/−^* mice and wild-type controls in their livers but not in their kidneys at 7 and 11 months of age.

**Table 3.**
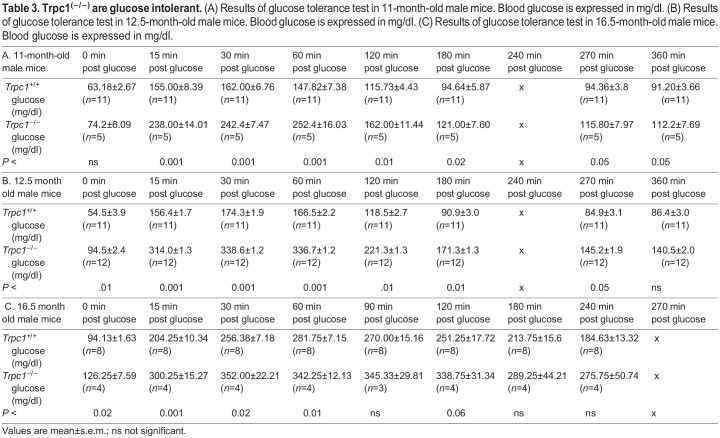
**Trpc1^(−/−)^ are glucose intolerant.** (A) Results of glucose tolerance test in 11-month-old male mice. Blood glucose is expressed in mg/dl. (B) Results of glucose tolerance test in 12.5-month-old male mice. Blood glucose is expressed in mg/dl. (C) Results of glucose tolerance test in 16.5-month-old male mice. Blood glucose is expressed in mg/dl.

B. 12.5 month old male mice	0 min post glucose	15 min post glucose	30 min post glucose	60 min post glucose	120 min post glucose	180 min post glucose	240 min post glucose	270 min post glucose	360 min post glucose
*Trpc1^+/+^* glucose (mg/dl)	54.5±3.9 (*n*=11)	156.4±1.7 (*n*=11)	174.3±1.9 (*n*=11)	166.5±2.2 (*n*=11)	118.5±2.7 (*n*=11)	90.9±3.0 (*n*=11)	x	84.9±3.1 (*n*=11)	86.4±3.0 (*n*=11)
*Trpc1^−/−^* glucose (mg/dl)	94.5±2.4 (*n*=12)	314.0±1.3 (*n*=12)	338.6±1.2 (*n*=12)	336.7±1.2 (*n*=12)	221.3±1.3 (*n*=12)	171.3±1.3 (*n*=12)	x	145.2±1.9 (*n*=12)	140.5±2.0 (*n*=12)
*P* <	.01	0.001	0.001	0.001	.01	0.01	x	0.05	ns

C. 16.5 month old male mice	0 min post glucose	15 min post glucose	30 min post glucose	60 min post glucose	90 min post glucose	120 min post glucose	180 min post glucose	240 min post glucose	270 min post glucose
*Trpc1^+/+^* glucose (mg/dl)	94.13±1.63 (*n*=8)	204.25±10.34 (*n*=8)	256.38±7.18 (*n*=8)	281.75±7.15 (*n*=8)	270.00±15.16 (*n*=8)	251.25±17.72 (*n*=8)	213.75±15.6 (*n*=8)	184.63±13.32 (*n*=8)	x
*Trpc1^−/−^* glucose (mg/dl)	126.25±7.59 (*n*=4)	300.25±15.27 (*n*=4)	352.00±22.21 (*n*=4)	342.25±12.13 (*n*=4)	345.33±29.81 (*n*=3)	338.75±31.34 (*n*=4)	289.25±44.21 (*n*=4)	275.75±50.74 (*n*=4)	x
*P* <	0.02	0.001	0.02	0.01	ns	0.06	ns	ns	x

### Homeostatic model assessment of insulin resistance (HOMA-IR)

Insulin resistance was confirmed by calculating HOMA-IR in the *Trpc1^−/−^* mice vs wild-type controls (4.6±1.4, *n*=12 vs 0.88±0.1, *n*=11 *P*<0.05). Insulin (17.7±4.3 micro-Units, *n*=12) was higher in *Trpc1^−/−^* mice than in wild-type controls (6.5±0.7 micro-Units, *n*=11). As HOMA-IR results vary considerably secondary to changes in beta cell function over time, we measured HOMA-beta cell function, which was consistently comparable between *Trpc1^−/−^* mice and wild-type control: 90±13%, *n*=12, vs 99±12%, *n*=11 not significant (ns) at 12 months and 85±16%, *n*=12, vs 93±15%, *n*=11, ns at 13 months. HOMA-beta formula was slightly modified to adapt it to the mice, using the same formula for both the wild-type mice and null mice. HOMA-IR was elevated in 4.5-month-old homozygous mutant males (*P*<0.0001) and females (ns) (Tables 1,2). Leptin level was not different and adiponectin level was mildly elevated in homozygous mutant mice, but it is difficult to interpret owing to the different number and size of adipocytes between genotypes (Tables 1,2). Indeed we found conflicting results at other ages (data not shown). C-peptide corroborated the elevated endogenous insulin levels in males. Glucagon was lower in the *Trpc1^−/−^* mice, as expected with the elevated blood glucose (Tables 1,2).

### Analysis of serum lipids

Lipid profile was measured at 12 and 22 months. Data confirmed a 30% increase in serum total cholesterol, 60% increase in serum LDL cholesterol, and 200% increase in serum triglyceride (TG) levels in *Trpc1^−/−^* mice compared with the wild-type controls, with *P*-values of <0.005, <0.005 and <0.005 respectively at 12 months. At 22 months, triglycerides in *Trpc1^−/−^* mice were increased by 50%, *P*<0.001 in *Trpc1^−/−^* mice, however cholesterol was not different (Table 4). This strengthens our hypothesis that metabolic syndrome can be created by the deletion of both copies of the *Trpc1* gene. Littermate *Trpc1^−/−^* mice compared to wild-type controls had elevated triglycerides at 12 months of age (data not shown), and at 18 months but serum cholesterol was elevated only at 18 months (Table 4). Both male and female *Trpc1^−/−^* mice had elevated serum triglyceride levels at 4.5 months and 13.5 months, but serum cholesterol was comparable to age-matched wild-type controls (Tables 1,2). The inconsistency in elevation of serum cholesterol is presently unexplained.

**Table 4.**
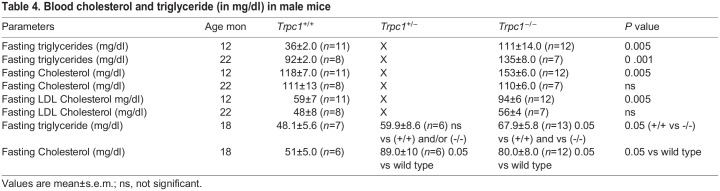
Blood cholesterol and triglyceride (in mg/dl) in male mice

### Liver echogenicity

Liver echogenicity in *Trpc1^−/−^* mice was increased as early as the 7th month of age compared to age-matched wild-type controls, consistent with hepatic steatosis. Repeat ultrasound at 11th month demonstrated worse hepatic steatosis with chronicity of the metabolic syndrome (Table 5). *Trpc1^−/−^* mice, *n*=32 similarly had increased echogenicity compared to 29 control littermates at 12 months (45.1±3.0 vs 34.9±1.8, *n*=29, *P*<0.01). At 12 months of age, liver density in heterozygous mice was 34.6, lower than *Trpc1^−/−^* mice, *P*<0.05, but not different from littermate wild-type controls. At 17 months, liver density was elevated in *Trpc1^−/−^* mice (61.0±4.5, *n*=13 vs 46.1±4.3, *n*=7 wild-type controls, *P*<0.05). In *Trpc1^+/−^* mice, liver density was intermediate in values (60.0±7, *n*=8), though not significantly different from either the wild-type controls or from the homozygous null mice (data not shown). Liver weights were 36% heavier in the *Trpc1^−/−^*mice, *n*=8, vs wild-type controls, *n*=7, (1.267±0.034 vs 0.933±0.041) at 9 weeks of age, *P*<0.001. Accordingly, the ratio of liver weight to body weight (mg/g) was 13% greater in *Trpc1^−/−^*mice (44.8±1.0) than the wild-type controls (39.7±1.0) (*P*<0.005). Total liver triglycerides were elevated in 2-month-old *Trpc1^−/−^*mice, 9.45±0.58 vs 6.4±0.68 mg/total liver, *P*<0.005. Liver triglyceride concentration (µg/mg of liver) was not different at this age. However, the increased liver weight accounted for the elevated total liver triglycerides. At 18 months, *Trpc1^−/−^*mice had elevated liver triglycerides (21.1±1.6 vs 11.0±3.7 mg triglyceride/g liver, *P*<0.04). Liver triglycerides in *Trpc1^+/−^* mice were 13.4±2.3, *P*<0.02 vs null, but not different from controls. Liver weights were not different in this group at this age; however, total liver triglyceride level was elevated by 107% (26.9±2.8 vs 13.01±5.1, *P*<0.05). In *Trpc1^+/−^* mice, total triglyceride level (17.4±6.0 mg/g liver) was intermediate between nulls and wild-type controls, but not different from either *Trpc1^−/−^*mice or wild-type controls (data not shown). Liver cholesterol, not shown, was not different (data not shown).

**Table 5.**
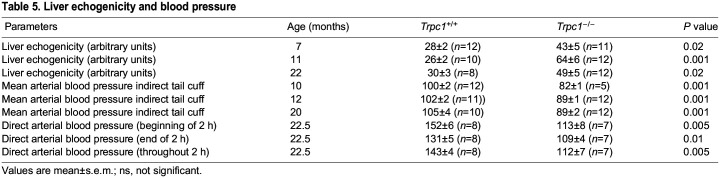
Liver echogenicity and blood pressure

### Systemic blood pressure

Hypertension is a part of the WHO criteria for metabolic syndrome. Our *Trpc1^−/−^*mice did not develop systemic hypertension. At 10, 12 and 20 months of age, mean arterial blood pressure measured by the tail-cuff method was in fact lower in *Trpc1^−/−^*mice (Table 5). On average, mean arterial blood pressure was 82 mm Hg in *Trpc1^−/−^*mice vs 100 mm of Hg in age-matched wild type control (*P*<0.001). Similar findings were obtained by direct blood pressure measurement via intra-arterial cannulation at the time of sacrifice (Table 5), confirming the absence of hypertension in *Trpc1^−/−^*mice. Though this is superficially at odds with the traditional or classical definition of metabolic syndrome, the absence of hypertension in the *Trpc1^−/−^* mice despite expression of other salient features of metabolic syndrome including obesity, glucose intolerance, insulin resistance, and hyperlipidemia, could be due to the lack of the principal or primary gene product(s) responsible for hypertension in *Trpc1^−/−^* mice. Indeed, studies have been published that show the close and parallel relationships between TRPC and hypertension (Schmidt et al., 2010; Kennedy et al., 2010).

### Hematocrit

*Trpc1^−/−^*mice had mild anemia in both genders (Tables 1,2), beginning at 2–3 months of age, in the absence of renal failure (Onopiuk et al., 2020) and through the last point of observation in our studies (13.5 months). The anemia may contribute to the absence of hypertension, although such an interpretation is at present conjectural without additional evidence (Cinar et al., 1999; Cirillo et al., 1992). Although conflicting, both anemia and elevated hematocrit have been observed in metabolic syndrome. (Feng et al., 2020; Huang et al., 2018; Kelem et al., 2023; Timerga et al., 2022; Wu et al., 2013)

## DISCUSSION

We have described a mouse phenotype that mimics metabolic syndrome, currently defined by the expression of a combination of three or more of the following five criteria: hyperinsulinemia, central obesity, dyslipidemia, hypertension, and hyperglycemia (Huang, 2009). Our *Trpc1^−/−^*mice demonstrate the following four features: increased body weight, elevated plasma glucose, increased plasma insulin, and hyperlipidemia. In fact, paradoxically, the *Trpc1^−/−^*mice had mild hypotension measured by both the indirect tail cuff method and by direct intra-arterial recordings. Lower blood pressure in *Trpc1^−/−^*mice has previously been found (Schmidt et al., 2010). Cases of metabolic syndrome without hypertension have also been reported in humans and in mice (Hong et al., 2015; Kennedy et al., 2010). The co-existence of several cardiovascular metabolic risk factors, including diabetes**,** abdominal obesity, and dyslipidemia, empirically observed in the phenotypes of the *Trpc1^−/−^*mice supports the hypothesis of metabolic syndrome. The TRPC superfamily comprises various cation-permeant channels shown to be involved in signal transduction, cell activation and metabolism. Some members have previously been implicated in or linked to obesity, dyslipidemia, and diabetes, several features often grouped under the name ‘metabolic syndrome’. Our findings support this concept. TRPC1 was the first TRPC channel cloned and identified in the pancreatic beta cells. Its expression has been shown to be reduced in diabetes (Zhang et al., 2009; Niehof and Borlak, 2008), although the precise relationship was unclear. Obesity, particularly abdominal obesity, is associated with resistance to the action of insulin on peripheral glucose and fatty acid use, often leading to type 2 diabetes mellitus. Insulin resistance, the associated increase in insulin, hyperglycemia, and the disturbed metabolism of adipocyte cytokines may also lead to vascular endothelial dysfunction, abnormal lipid profile, and vascular inflammation, all of which predispose the individual to the development of cardiovascular diseases and/or the metabolic syndrome, the deadly quartet, or the obesity dyslipidemia syndrome. Thus it has been proposed that the TRPC channels could play a key role in the metabolic syndrome. Our present results are consistent with this hypothesis. After all, it has recently been shown that constitutively active TRPC channels of adipocytes confer a mechanism for sensing dietary fatty acids and regulating adiponectin (Sukumar et al., 2012). Protein kinase C (PKC) promotes insulin secretion via TRPC phosphorylation in INS-1E cells (Xu et al., 2019). Reduced expression of TRPC1 has been reported in diabetic nephropathy (Niehof and Borlak, 2008). Here, we tested the hypothesis raised by Liu et al. (2008) that homozygous TRPC gene deletion could produce metabolic syndrome in mice. Results of our current experiments in TRPC-null mice would support this hypothesis.

The role of TRPC1 in adiponectin secretion, body composition and adipose tissue has been studied extensively and TRPC1 was found to play a key role (Schaar et al., 2019; Schaar, 2018). TRPC1 was shown to regulate brown fat tissue activity (Wolfrum et al., 2018). It has been proposed that leptin excites pro-opio-melanocortin neurons through TRPC1 channels (Qui et al., 2010; Perissinotti et al., 2021). Cytokine leptin and leptin receptors are both involved in appetite regulation. This could account for the increased appetite observed in our *Trpc1^−/−^* mice.

### *Trpc1^−/−^*mice

The homeostatic model assessment of insulin resistance (HOMA-IR) and beta cell function (HOMA-beta) were reported in 1985 (Matthews et al., 1985) to distinguish between insulin resistance and insulin deficiency from beta cell defects. Limitations of the homeostatic model have been discussed (Kang et al., 2005; Wallace et al., 2004). This model is however validated in rats (Antunes et al., 2016). It assumes beta cell function of 100% and insulin resistance of 1. Mice often have a fasting glucose level lower than that in humans; we therefore modified the formula for calculating beta cell function in mice but applied the same formula to both wild-type and *Trpc1^−/−^* mice. HOMA-IR in *Trpc1^−/−^*mice indicated they had insulin resistance and HOMA-beta cell function showed no differences.

Nonalcoholic liver disease (NASH) is a common cause of chronic liver disease (Ogasawara et al., 2011), making it ideal if not necessary to have animal models to study this syndrome. In NASH, excess fat accumulates in the liver (Takahashi et al., 2012). Liver fatty acids have been analyzed in *Trpc1^−/−^*mice. The liver profile was reported to be similar to an obese phenotype (Schaar, 2018). Livers in our *Trpc1^−/−^*mice were more echogenic. We found increased liver weight and increased ratio of liver / body weight. In addition, *Trpc1^−/−^*mice had increased liver triglyceride concentration at sacrifice and increased total liver triglycerides. Ca signaling has been reviewed and proposed as a therapeutic target in NASH (Ali and Petrovsky, 2019).

Calcium ion plays a pivotal role in various cellular processes including muscle contraction, release of transmission signals, cell proliferation, gene transcription, and even cell demise. Our studies suggest that the absence of TRPC channels could create the condition of metabolic syndrome. TRP channels play an important role in the regulation of cytosolic free Ca^2+^ either by acting as Ca^2+^ entry pathways in the plasma membrane, or by changes in membrane potential, curbing the driving force for Ca^2+^ entry mediated by alternative pathways. TRP channels also form intracellular pathways for Ca^2+^ release from several cell organelles. Given the exceptional importance of Ca^2+^ signaling in virtually all cell processes, it is not surprising that dysfunctions in Ca channels are causal to, or at least involved in, the pathogenesis of several disease disorders. We recently described *Trpc1^−/−^*mice presenting a syndrome similar to that of familial hypocalciuric hypercalcemia (FHH) in man (Onopiuk et al., 2020; Eby et al., 2022). However until our experiments, with respect to the TRP channels, there are at present only a few conditions that can be strictly qualified as “channelopathy”, in which a defect in channel function is the established or proven direct or unequivocal cause of the disease. In general, TRPC members can be considered as channels activated subsequent to stimulation of receptors that activate different isoforms of phospholipase C (PLC)-beta. TRPC3, −6, and −7 are galvanized by diacylglycerol (DAG), independent of the stimulation of protein kinase C, suggesting that DAG mediates their physiological activation. In contrast, TRPC1, −4, and −5, which are also activated by receptor-induced PLC, are mostly unresponsive to DAG. However, the mechanism by which PLC stimulation leads to activation of these channels remains poorly defined.

## MATERIALS AND METHODS

### Animals

Age-matched male *Trpc1^+/+^* mice and *Trpc1^−/−^*mice on pure 129S/SvEv backgrounds were obtained, respectively, from Taconic Farms (Germantown, NY, USA) or Jackson Labs (Bar Harbor, ME, USA) or in-house from controls breeder pairs obtained from Taconic Farms and as a gift from Lutz Birnbaumer (Liu et al., 2007). They were studied from the 7th to 23rd months following established methods (Lau and Eby, 1982; He et al., 2017; Reininger et al., 2010). To evaluate the possible effect of differential food intake by pregnant and nursing dams between different genotypes on perinatal growth and to exclude this as a confounding variable, litter mates *Trpc1* wild-type (*Trpc1^+/+^* mice), *Trpc1* heterozygote (*Trpc1^+/−^*mice) and *Trpc1-*null (*Trpc1*^−/−^mice) mice, bred from the same hetero-zygotic parents and nursed by the same hetero-zygotic dams, were also studied. These littermate progenies were studied from 1st–18th month. All mice were housed in IACUC-approved facility with 12 h:12 h day:night cycles and fed regular mouse chow (Purina) and tap water *ad libitum*. Serial body weights were monitored weekly. Food and fluid intakes were evaluated in individual metabolic cages while undergoing a 24-h urine collection as a part of the metabolic studies. Tail blood, fasted or non-fasted as appropriate, was obtained at designated times. The protocols were reviewed and approved by University of Oklahoma Health Sciences IACUC committee (IACUC # 14-11-NSR, 10-170-H, 08-0050H and 04-159-R).

### Glucose tolerance tests

Blood glucose was measured by glucose strips (ReliOn Ultima blood glucose strips, ABBOTT DIABETES CARE) and insulin by mouse enzyme-linked immunosorbent assay (ELISA) (Alpco, Crystal Chem). A 6 h glucose tolerance test was performed after a 16 h overnight fast at 7:00–8:00 AM and by an intraperitoneal injection of 2 mg glucose per g body weight as previously described (Andrikopoulos et al., 2008). HOMA1-IR and HOMA-beta cell functions were calculated based on established formulae (Ascaso et al., 2003; Festa et al., 2008).

### Blood analysis

Cholesterol and triglycerides were analyzed enzymatically (Thermo Fisher Scientific) by plate readers. Adiponectin, leptin, C-peptide, glucagon and insulin were measured using corresponding ELISA kits obtained from Crystal Chem (USA).

### Blood pressure

Blood pressure was measured by the tail cuff method and confirmed later after euthanasia by direct intra-arterial monitoring via an indwelling catheter, as previously described (Lau and Eby, 1982; He et al., 2017; Reininger et al., 2010; Xie et al., 2011). In the 9th month, mice were trained to get systolic and diastolic blood pressures measured by the tail cuff method using a computerized automated system dependent on a laser sensor Visitech Systems blood pressure 2000 tail cuff system (Visitech Systems, NC, USA). Mean blood pressure was calculated as [(2×diastolic)+systolic]/3 since 2/3 of the cardiac cycle is diastole. Tail cuff blood pressure was measured at 10, 12 and 20 months. When euthanized at 22.5 months, under pentobarbital anesthesia, tracheostomy was performed, followed by cannulation of the carotid artery for blood sampling and for intra-arterial blood pressure measurements using a Hewlett Packard pressure transducer as previously described (Lau et al., 1986a,b).

### Ultrasound

Liver and kidney density were studied by ultrasound (Philips Agilent technology ultrasound system) with a 15 L-8 MHz transducer, in mice which were lightly anesthetized by using a mixture of 1.5% isoflurane and 0.5 L/min O_2_ (Xei et al., 2011;Pollard et al., 2006; Benjamin et al., 2017; Han et al., 2014). To optimize sound conduction, skin hair was removed with depilatory cream. Images were obtained from longitudinal and transverse sections of both the right and left kidneys in each animal. Echogenicity was determined on a longitudinal image including the right kidney and liver. Density was measured using Image J software (Schneider et al., 2013). Liver density was determined at 7, 11, and 22 months in age-matched males and also in 12- and 17-month-old littermates.

### Liver lipids

At sacrifice, the livers were removed and total wet organ weights were obtained. Weights were expressed as both total organ weight and as organ weight per g body weight. Organs were stored at −80°C until analyzed. Triglycerides and cholesterol were extracted from weighed pieces of minced liver, using chloroform: methanol extraction as previously described (Bligh and Dyer, 1959). Triglycerides and cholesterol were measured enzymatically (Thermo Fisher Scientific).

### Statistical analysis

All results are presented as mean±standard error of the mean (s.e.m.) and were compared using Students’ *t*-test or analysis of variance (ANOVA), as appropriate.
